# Whole-body vibration as a mode of dyspnoea free physical activity: a community-based proof-of-concept trial

**DOI:** 10.1186/1756-0500-6-452

**Published:** 2013-11-11

**Authors:** Trentham Furness, Corey Joseph, Liam Welsh, Geraldine Naughton, Christian Lorenzen

**Affiliations:** 1 School of Nursing, Midwifery and Paramedicine, Australian Catholic University, Fitzroy, Australia; 2 School of Exercise Science, Australian Catholic University, Fitzroy, Australia; 3 NorthWestern Mental Health, The Royal Melbourne Hospital, Grattan Street, Parkville, Victoria 3050, Australia; 4 Centre for Sports and Exercise Medicine, Queen Mary University, London, UK; 5 Royal Children’s Hospital, Melbourne, Australia

**Keywords:** Dyspnoea, Whole-body vibration, Heart rate, Acute responses

## Abstract

**Background:**

The potential of whole-body vibration (WBV) as a mode of dyspnoea free physical activity for people with chronic obstructive pulmonary disease (COPD) is unknown among community-based settings. Furthermore, the acute effects of WBV on people with COPD have not been profiled in community-based settings. The aim of this community-based proof-of-concept trial was to describe acute effects of WBV by profiling subjective and objective responses to physical activity.

**Findings:**

Seventeen community-dwelling older adults with COPD were recruited to participate in two sessions; WBV and sham WBV (SWBV). Each session consisted of five one-minute bouts interspersed with five one-minute passive rest periods. The gravitational force was ~2.5 *g* for WBV and ~0.0 *g* for SWBV. Reliability of baseline dyspnoea, heart rate, and oxygen saturation was first established and then profiled for both sessions. Acute responses to both WBV and SWBV were compared with repeated measures analysis of variance and repeated contrasts. Small changes in dyspnoea and oxygen saturation lacked subjective and clinical meaningfulness. One session of WBV and SWBV significantly increased heart rate (*p* ≤ 0.02), although there was no difference among WBV and SWBV (*p* = 0.67).

**Conclusions:**

This community-based proof-of-concept trial showed that a session of WBV can be completed with the absence of dyspnoea for people with COPD. Furthermore, there were no meaningful differences among WBV and SWBV for heart rate and oxygen saturation. There is scope for long-term community-based intervention research using WBV given the known effects of WBV on peripheral muscle function and functional independence.

## Findings

### Background

A disease of the lungs, chronic obstructive pulmonary disease (COPD) is a leading cause of mortality worldwide [1]. Data of the Australian Bureau of Statistics report COPD to be more common than most cancers, road traffic accidents, coronary artery disease, and diabetes [2]. An estimated 2.1 million Australians are affected by COPD, with an estimated increase to 4.5 million by 2050 [2]. Considering the known effect of COPD on mortality and a predicted increase of incidence of COPD, research about assessment, prevention, management, and treatment of the disease is salient.

Peripheral muscle training is an important component of pulmonary rehabilitation for the management of COPD [3]. Position statements from respiratory bodies including the GOLD, the American Thoracic Society and the European Respiratory Society support the use of resistance training and aerobic conditioning as modes of physical activity to improve peripheral muscle function of people with COPD. The risk of acute dyspnoea or hypoxemia during resistance training [4,5] and or aerobic conditioning [6-8] is secondary to the known risks associated with physical inactivity. An ongoing challenge of peripheral muscle training therefore, remains to design physical activity interventions that stimulate the cardiovascular and musculoskeletal systems to allow physiological adaptations yet maximise compliance [9].

Whole-body vibration (WBV) is a mode of physical activity known to strengthen the peripheral muscles of the lower limbs [10,11]. A growing body of literature on the effects of WBV within sub-optimal health populations exists, yet studies of effects of WBV on people with COPD are limited in number and confined to the hospital/laboratory. Despite the potential benefits of WBV to strengthen peripheral muscles of the lower limbs when incorporated with pulmonary rehabilitation, the acute responses of people with COPD to WBV have not been profiled in community-based settings. As such, this proof-of-concept trial was conducted to determine if WBV could be a mode of dyspnoea free physical activity and was the first stage of community-based Phase II efficacy trial [12]. The aim of this trial was to profile acute subjective and objective responses of people with COPD to a single session of WBV and a single session of sham WBV (SWBV). If as a concept, WBV could be completed with the absence of dyspnoea, compliance to a long-term community-based WBV intervention may be enhanced among people with COPD. As such, it was hypothesised there would be no difference of perceived dyspnoea, heart rate, and oxygen saturation among WBV and SWBV.

## Methods

Seventeen adults with COPD provided informed voluntary consent to participate in the trial. The trial was approved by the Southern Health Human Research Ethics Committee A. The trial was registered with the Australian New Zealand Clinical Trials Registry (ANZCTR12612000508875). Participants were recruited from Metropolitan Melbourne and the Mornington Peninsula, Victoria, Australia. Data were collected in the home of each participant by TF.

Participants were community-dwelling and functionally independent. Spirometry data were collected as part of usual care at the Monash Medical Centre with the methods described by American Thoracic Society and the European Respiratory Society and GOLD classifications.

The subjective dependent variable of perceived dyspnoea was quantified with the Borg category-ratio CR-10 (Borg CR-10) [13] visual analogue scale. The objective dependent variables were heart rate and oxygen saturation (SpO_2_), quantified using the CARESCOPE™ V100 Vital Sign Monitor (GE Health Care, Milwaukee, USA). Reliability of the dependent variables was established prior to the WBV and SWBV sessions across a standard test-retest-retest protocol [14] in the home of each participant. Intraclass correlation coefficients were classified as 'acceptable’ (ICC ≥ 0.700) for all dependent variables.

Participants first completed a single session of WBV, then a single session of SWBV at least seven days later. In accordance with our trial protocol [12], this proof-of-concept trial was completed as a non-randomised intervention with crossover to sham. A side alternating vibration platform (Amazing Super Health, Melbourne, AUS) was used. For the single WBV session, platform frequency was ~25 Hz and peak-to-peak displacement was ~2.0 mm, peak acceleration was ~24.7 m.s^-2^, gravitational force was ~2.5 *g*. For the single SWBV session, platform frequency was ~25 Hz and peak-to-peak displacement was ~0.0 mm. Foot placement (second toe) was equidistant, 20 cm from the axis of rotation for both WBV and SWBV. Participants wore flat soled shoes and stood with ~20° knee flexion when checked with a manual goniometer during each session. The single WBV and SWBV sessions consisted of five, one-minute vibration bouts, interspersed with one-minute of passive rest. Although the most effective test protocol is yet to be established, the selected test protocol had been used previously to describe acute effects of WBV among other populations [15-17]. During rest, the participant remained on the vibration platform, but was encouraged to stand with a posture that mimicked the 'anatomical position’ (International Society for the Advancement of Kinanthropometry). Skidding was checked to confirm the vibration platform gravitational forces with the methods of the International Society of Musculoskeletal and Neuronal Interactions [18].

Data were collected at baseline (after the participant had been sitting quietly in a chair for five minutes) and within 30 seconds of the completion of the final WBV or SWBV bout. After normality, repeated measures analysis of variance with repeated contrasts were computed for differences between rest and during a bout (n = 17) with SPSS (Version 19, Chicago, IL). Effect size was reported as Partial eta squared (Partial η^2^). To minimise the potential for familywise error rate, significance was accepted at *p* ≤ 0.02.

## Results

With GOLD classification, 14 participants were affected by moderate Stage II COPD and three participants by severe Stage III COPD (Table 1). Changes in perceived dyspnoea remained stable from 'very slight’ to 'slight’ among WBV and SWBV (Table 2). Both single sessions increased heart rate (*p* = 0.01). The increase in heart rate was more with WBV (11 beats.min^-1^, Partial η^2^ = 0.88) than with SWBV (8 beats.min^-1^, Partial η^2^ = 0.70). The difference among heart rate increases was not significant (*p* = 0.67). Oxygen saturation reduced by 1% during WBV (*p* = 0.08) and did not change during SWBV (*p* = 0.61).

**Table 1:** Descriptors of the participants with COPD

**Descriptor**	**Pre**			**Post**		
	**Mean**	**SD**	**95% CI**	**Mean**	**SD**	**95% CI**
FEV_1_ (L BTPS)	1.4	0.5	± 1.0	1.6	0.6	± 1.2
FEV_1_ % predicted	52.1	17.5	± 34.3	58.2	19.4	± 38.0
FVC (L BTPS)	2.8	0.8	± 1.6	3.1	0.8	± 1.6
FVC % predicted	80.3	16.1	± 31.6	84.5	17.3	± 33.9
FER %	52.0	13.7	± 26.9	52.7	11.8	± 23.1
PEF (L.sec^-1^)	3.8	1.4	± 2.7	4.2	0.9	± 1.8
Age (years)	69	8				
Stature (m)	1.7	0.9				
Mass (kg)	83.9	19.2				
BMI (kg.m^-2^)	24.7	5.1				
SBP (mmHg)	136	15				
DBP (mmHg)	73	11				

BMI: body mass index. SBP: systolic blood pressure. DBP: diastolic blood pressure.

**Table 2:** Effects of WBV and SWBV on subjective and objective variables

	**WBV**				**SWBV**			
**Dependent variable**	**Mean (SD)**	**95% Confidence (SE)**		**Sig. (Partial eta squared)**	**Mean (SD)**	**95% Confidence (SE)**		**Sig. (Partial eta squared)**
		**Lower**	**Upper**			**Lower**	**Upper**	
Borg CR-10 baseline	1 (2)	0	2	0.01*	1 (1)	1	2	0.08
Borg CR-10 during bout	2 (2)	1	3	(0.50)	2 (1)	1	3	(0.36)
HR baseline (beats.min^-1^)	81 (12)	75	87	0.01*	85 (12)	79	91	0.01*
HR during bout (beats.min^-1^)	92 (13)	85	99	(0.88)	93 (14)	86	100	(0.70)
SpO_2_ baseline (%)	97 (2)	96	97	0.08	95 (2)	95	96	0.61
SpO_2_ during bout (%)	96 (2)	95	97	(0.18)	95 (2)	95	97	(0.02)

*; *p* ≤ 0.02.

## Discussion

Findings of this community-based proof-of-concept trial support the use of WBV as a mode of dyspnoea free physical activity for people with COPD. Furthermore, there were no meaningful differences among WBV and SWBV for acute subjective and objective variables.

The results show WBV does not elicit a physical demand comparable with resistance training and aerobic conditioning for people with COPD. People with stage II COPD perceived dyspnoea (Borg CR-10) less during WBV compared with resistance training [4], treadmill walking [19], and the six-minute walk test [20]. Heart rate during WBV in this community-based trial was lower compared with combined resistance training and aerobic conditioning [4] and fast paced exercise [7]. In this community-based trial, oxygen saturation reduced from 97 to 96% during WBV, yet the clinical meaningfulness was negligible due to an absence of hypoxemia despite the moderate effect size (Partial η^2^ = 0.18) (Table 2). Safety recommendations are that exercise should be terminated if oxygen saturation reduces to ≤ 85% [21,22].

The WBV gravitational force of the vibration platform in this community-based trial was ~2.5 *g*. For non-community dwelling older adults, heart rate increased to 135 beats.min^-1^ when the gravitational force was 9.5 *g*[17]. Exposure to WBV at 8.0 *g* in healthy adults elevated heart rate reserve by 8% [23]. However, the potential for higher heart rates with increased gravitational forces during WBV should be offset with the risk of bone damage [24]. Subsequently, the appropriateness of prescribed doses of WBV should be at the forefront of exercise intervention planning for people with COPD.

The results of this community-based proof-of-concept trial support the use of WBV as a mode of dyspnoea free physical activity for people with COPD. However, not every prescribed load and protocol will be appropriate and dyspnoea responses may vary depending on desensitisation. Further study of WBV among clinical setting may describe acute effects on dyspnoea in patients with 'severe’ COPD. Among community-based settings, further study of effects of WBV on peripheral muscle function of the lower limbs should be conducted to establish efficacy of WBV as a strengthening tool because the results of this trial support the use of WBV as a dyspnoea free mode of physical activity for people with COPD.

## Conclusions

Using dose appropriate prescription, WBV was a mode of dyspnoea free physical activity for people with COPD in this community-based proof-of-concept trial. Acute changes in subjective and objective responses to physical activity during WBV were clinically meaningless. The potential of long-term community-based WBV interventions to improve function of the peripheral muscles of the lower limbs remains unknown yet should be described because WBV did not change perceived breathlessness of people with COPD.

## Competing interests

The authors declare they have no competing interests.

## Authors’ contributions

All authors made substantial contribution to the conception and design of the trial, and preparation of this manuscript. TF recruited all participants, coordinated the trial, collected, and analysed all data. All authors give final approval of this version to be published.
